# Visual and refractive outcomes of opposite clear corneal incision combined with rotationally asymmetric multifocal intraocular lens implantation

**DOI:** 10.3389/fmed.2024.1389186

**Published:** 2024-06-28

**Authors:** Xiaoyu Qin, Pengxiang Yao, Xinyuan Wu, Yang Wu, Yufang Hong, Zhenzong Chen, Yuanzhi Yuan

**Affiliations:** ^1^Department of Ophthalmology, Zhongshan Hospital (Xiamen), Fudan University, Xiamen, China; ^2^Department of Ophthalmology, Zhongshan Hospital, Fudan University, Shanghai, China; ^3^The Centre for Evidence-based Medicine, Fudan University, Shanghai, China

**Keywords:** corneal curvature, astigmatism, cataract surgery, incision position, multifocal intraocular lens

## Abstract

**Purpose:**

To evaluate the visual and refractive outcomes of astigmatic cataract patients following opposite clear corneal incision (OCCI) combined with rotationally asymmetric multifocal intraocular lens (IOL) implantation.

**Setting:**

Department of Ophthalmology, Zhongshan Hospital (Xiamen), Fudan University, People’s Republic of China.

**Design:**

Retrospective cohort study.

**Methods:**

This study comprised 58 cataract eyes of 54 patients with corneal astigmatism who underwent phacoemulsification and rotationally asymmetric multifocal IOL implantation which received either OCCI (OCCI group) or a single clear corneal incision (SCCI group). The follow-up period was 3 months after surgery. Distance, intermediate and near visual acuity, refractive outcomes, and corneal anterior keratometry were compared between the two groups. Vector analysis was used to evaluate astigmatism correction.

**Results:**

Three months after surgery, the distance, intermediate and near visual acuity, and sphere remained comparable between the two groups, but a significant difference was detected in residual astigmatism and anterior corneal keratometric astigmatism. In the OCCI group, the residual astigmatism and keratometric astigmatism were −0.60 ± 0.29 D and 0.59 ± 0.28 D, respectively, which were lower than those in SCCI groups (−1.18 ± 0.47 D and 1.15 ± 0.45 D, both *p* < 0.05). In vector analysis, the difference vector (DV), angle of error (AoE), absolute AoE, index of success (IoS) and correction index (CI) were statistically significantly different between the two groups (*p* < 0.05).

**Conclusion:**

OCCI combined with rotationally asymmetric multifocal intraocular lens implantation showed predictable and desirable efficacy in treating cataract patients with astigmatism.

## 1 Introduction

Cataract is a leading cause of visual disability in the elderly. With the advances in the development of multifocal intraocular lenses (IOLs), the goal of contemporary cataract surgery is shifting toward achieving desirable refractive outcomes and visual quality, rather than simply restoring vision (1, 2). By generating several foci at different distances, multifocal IOLs are designed to reduce the dependence on spectacle after surgery by restoring distance, intermediate, and near vision. The traditional concentric refractive, diffractive, or a combination of refraction and diffraction multifocal IOLs, however, trade off the image quality for spectacle independence. Because the incoming light rays are split to produce 2 or more focal points, patients implanted with those IOLs can experience decreased contrast sensitivity, glare disability, halos, and other optical phenomena (3). Refractive rotationally asymmetrical multifocal IOLs have been introduced to reduce such side effects (4, 5). The design has been shown to improve visual outcomes and provide good patient satisfaction (6–8).

Corneal astigmatism is another refractive error that should be managed to achieve postoperative emmetropia and spectacle-free. We have demonstrated that nearly half of the cataract surgery candidates have corneal astigmatism ≥ 1.00 D, and the magnitude of astigmatism increases with age (9). Meanwhile, 78% of cataract patients over the age of 65 had astigmatism greater than 0.5D (10). According to the EUREQUO database, even for those whose postoperative absolute mean biometry prediction error (spherical equivalent refraction, SEQ) was no more than 0.5 D, 21.2% of them have postoperative cylinder more than 1.0 D (11). Studies of Steven and Shen provided that even low levels of residual astigmatism can degrade visual acuity (12, 13). Therefore, reliable corneal astigmatism control or correction in cataract surgery is critically important for better visual outcomes and patient satisfaction. There are various options for the reduction of corneal astigmatism during cataract surgery, including the position selection of cataract incision, peripheral corneal relaxing incisions, Toric intraocular lens implantation, intrastromal ring implantation and surface ablation (14–16).

Opposite clear corneal incision (OCCI) involves a pair of incisions in the clear cornea that flattens the corneal curvature in that meridian (17). Studies have shown, in terms of reducing postoperative astigmatism, that the procedure is effective and safe in combination with spherical or aspherical monofocal IOL implantation (18–20). However, it was not clear whether OCCI with rotationally asymmetrical multifocal IOL implantation can improve the optical and visual outcomes for cataract patients with astigmatism, thus far achieving the goal of spectacle-free.

In this study, we aim to investigate the efficacy and safety of the technique by comparing the visual and refractive outcomes of a group of cataract patients with or without OCCI during phacoemulsification and rotationally asymmetric multifocal IOL implantation.

## 2 Patients and methods

### 2.1 Patient selection

In this study, patients received phacoemulsification and rotationally asymmetric multifocal intraocular lens implantation (Lentis Comfort LS-313 MF15, TELEON GmbH, Berlin, Germany) from 1 December 2022 to 30 April 2023 at Zhongshan Hospital (Xiamen), Fudan University were reviewed. The inclusion criteria were as follows: (1) Age 50 years or older; (2) Preoperative magnitude of manifest astigmatism ≥ 0.5D; (3) Preoperative magnitude of corneal astigmatism between 0.50 and 3.00 D; (4) Preoperative corrected distance visual acuity (CDVA) was 1.0 LogMAR or better.

The exclusion criteria were as follows: (1) patients with concomitant ophthalmic or systemic diseases affecting visual acuity, such as macular degeneration, retinal vascular diseases, glaucoma, or neurological disease; (2) missing key data on the pre- or post-operative measurement.

In total, 58 eyes of 54 patients were enrolled in this study. These patients were separated into two groups based on whether they underwent the OCCI or single clear corneal incision (SCCI) surgical procedure. The study was conducted in agreement with the tenets of the Declaration of Helsinki and was approved by the Review Board of Zhongshan Hospital (Xiamen), Fudan University (B2023-106R).

#### 2.1.1 Perioperative management

All patients underwent bio-measurement (IOL-Master 500, Carl Zeiss Meditec.AG) and had their IOL target refraction calculated by Barrett Universal II Formula preoperatively.^1^ Levofloxacin eye drops (Ofloxacin, Santen) were used 4 days prior to surgery. Following surgery, tobramycin dexamethasone eye drops and cream (Tobradex, Alcon) were used to provide anti-microbial and anti-inflammatory action for two weeks.

### 2.2 Surgical technique

All surgeries were performed by an expert surgeon (Dr. Yao P). Proparacaine hydrochloride eye drops (Alcaine, Alcon) were administered to induce surface anesthesia. The surgical procedures differed between the two groups when performing corneal incisions, as described below.

In the OCCI group, limbal marks were made before surgery at 3 and 9 o’clock positions on corneal while patients sitting in front of the slit lamp and looking straightly ahead to avoid cyclotorsion. Patients underwent topography examination (OCULUS 77000) before surgery, and the axis position of maximum keratometry from axial map was selected as main incision position.

**FIGURE 1:**
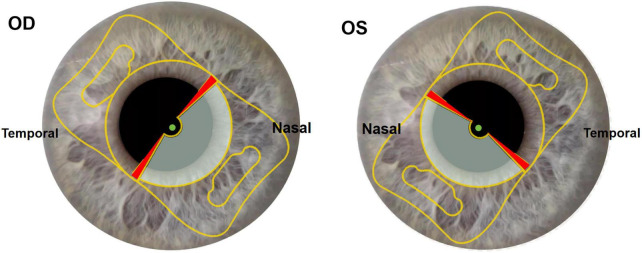
Slit lamp sample images of multifocal IOL implantation position. Gray part of optical zone: near vision function area. Transparent part of optical zone: distance vision function area. Red part of optical zone: transition region. Green point: intersection of visual axis and corneal anterior surface located in distance vision function area. OD: right eye. OS: left eye.

The main incision was performed at the crosspoint of the steep K axis and corneal limbal near the right hand (2.4 mm in width). An assisted incision was performed beside the main incision near the left hand (1 mm in width). Viscoelastic material was injected from the assisted incision to maintain the anterior-chamber depth. Continuous central circular capsulorhexis (approximately 5.5 mm in diameter) was performed. Hydrodissection and phacoemulsification were performed to remove the lens nucleus (Centurion, Alcon), followed by aspiration of the cortex. The IOL was implanted in the capsular bag through main incision using a Viscoject Bio 2.2 injector (Medicel AG, Altenrhein, Switzerland). The position of the intraocular lens (IOL) was adjusted by using a hook to ensure that the distance vision function area of the IOL was aligned with the optical axis, while the near vision function area was positioned at the sub-nasal region (Figure 1). The OCCI was made on the opposite position of the main incision at steep meridian using the same knife (2.4 mm in width). The irrigation-aspiration system was used to remove residual cortex. Finally, only the phacoemulsification incision and the assisted incision were hydrated at the end of the surgery.

In the SCCI group, limbal marks were not required. The main and assisted incisions were made at the sites of 11 and 1 o’clock (2.4 and 1 mm in width, respectively). The remaining surgical procedures were identical to those of the OCCI group, excluding the OCCI.

### 2.3 Preoperative and postoperative evaluations

Before surgery, all patients underwent measurements of corrected distance visual acuity (CDVA), manifest refraction, and keratometric astigmatism (IOL-master 500, Carl Zeiss Meditec AG). Three months post-surgery, in addition to reassessing CDVA, manifest refraction and keratometric astigmatism, we further evaluated uncorrected distance visual acuity (UDVA) and uncorrected near visual acuity (UNVA). All visual acuity outcomes were analyzed and reported in LogMAR units. The distance for near visual acuity was set at 30 cm and 60 cm. The changes of manifest refraction astigmatism and keratometric astigmatism were analyzed by using vector analysis method to assess the efficacy of astigmatism correction (21). Astigmatism was divided into three types according to cylinder axis: with the rule (WTR), against the rule (ATR) and obilque. WTR was defined as a negative cylinder from 0° to 29° or 151° to 180°, ATR between 90° ± 29° (61° and 119°), oblique between 120° and 150° and 30° and 60°. Astigmatism data was converted from the spectacle plane to the corneal plane.

Vector analysis was performed by ASSORT calculator,^2^ which was provided by Dr. Alpins in the official website of International Society Refractive Surgery (ISRS). The parameters of vector analysis were calculated as follows:

Target induced astigmatism (TIA): the astigmatism change that was supposed to be induced by surgery.
Surgically induced astigmatism (SIA): the astigmatism change that was induced actually by surgery.
Difference vector (DV): the vector difference of TIA and SIA.
Magnitude of error (ME): the arithmetic difference of SIA and TIA.
Angle of error (AoE): the angle difference of axis between TIA and SIA.
Correction index (CI): the ratio of SIA to TIA, this parameter could notice an astigmatic undercorrection or overcorrection when CI < 1 or > 1.
Index of success (IoS): the ratio of DV and TIA. A result of astigmatism correction was more desirable when IoS was closer to 0.

### 2.4 Statistical analysis

All data analysis were performed by using SPSS Statistic (version 27, IBM Corp). The normality of all data was first checked by Kolmogorov-Smirnov test. Normally distributed data was compared using the independent Student’s *t*-test, and non-normally distributed data was analyzed by Mann-Whitney U-test. A *p*-value less than 0.05 was considered statistically significant.

## 3 Results

### 3.1 Basic information

Fifty-four patients (58 eyes) were enrolled in this study, including 26 females (48.1%). The patients’ age was 69.8 ± 8.8 (range 51–86) years old. The SCCI group consisted of 28 patients (30 eyes), with 15 females (53.6%), and the age was 70.2 ± 7.4 (range 54–86) years old. The age of the 26 patients (28 eyes) in the OCCI group was 69.3 ± 10.1 (range 51–83) years old, with 11 females (42.3%). The age (*t* = −0.38, *p* = 0.71) and gender (*X*^2^ = 1.04, *p* = 0.71) were balanced across the two groups. Differences in axial length, CDVA, manifest refraction outcomes, keratometric astigmatism and scotopic pupil diameter between the two groups were comparable (Table 1). All surgeries were performed uneventfully, and no complications were observed during or after the procedure.

**TABLE 1:** Preoperative visual acuity and biometrical measures of the OCCI and SCCI groups.

Parameter	OCCI group (*n* = 28)	SCCI group (*n* = 30)	*p*
**LogMAR CDVA**			
Mean ± SD	0.40 ± 0.24	0.44 ± 0.17	0.23
Range	0.10, 0.80	0.15, 0.60	
**scotopic pupil (mm)**			
Mean ± SD	3.81 ± 0.54	3.87 ± 0.45	0.65
Range	3.10, 4.80	3.20, 4.80	
**Axial length (mm)**			
Mean ± SD	23.32 ± 0.79	23.32 ± 0.68	0.98
Range	21.71, 25.36	22.17, 24.58	
**Sphere (D)**			
Mean ± SD	−0.24 ± 1.93	0.12 ± 1.80	0.50
Range	−4.75, 3.50	−4.50, 3.50	
**Cylinder (D)**			
Mean ± SD	−1.04 ± 0.50	−1.12 ± 0.47	0.41
Range	−2.00, −0.50	−2.00, −0.50	
WTR	17 (60.70%)	19 (63.33%)	
ATR	8 (28.57%)	6 (20.00%)	
OA	3 (10.7%)	5 (16.67%)	
**Keratometric astigmatism (D)**			
Mean ± SD	1.19 ± 0.32	1.27 ± 0.46	0.89
Range	0.56, 1.75	0.64, 2.75	

OCCI, opposite clear corneal incision; SCCI, single clear corneal incision; CDVA, corrected distance visual acuity; SD, standard deviation; WTR, with the rule; ATR, against the rule; OA, oblique astigmatism.

### 3.2 Visual and refractive outcomes

Three months after surgery, no complications were detected in either group. The UDVA, CDVA, 60 cm-UNVA, and 30 cm-UNVA were 0.22 ± 0.18, 0.11 ± 0.11, 0.27 ± 0.16, 0.48 ± 0.17 separately in the OCCI group, and 0.20 ± 0.10, 0.12 ± 0.10, 0.26 ± 0.68 and 0.43 ± 0.78 in the SCCI group, respectively. There was no statistically significant difference in these measures between the two groups (Table 2).

**TABLE 2:** Postoperative visual acuity and biometrical measures of the OCCI and SCCI groups.

Parameter	OCCI group	SCCI group	*P*
**LogMAR UDVA**			
Mean ± SD	0.22 ± 0.18	0.20 ± 0.10	0.75
Range	0.00, 0.50	0.00, 0.40	
**LogMAR CDVA**			
Mean ± SD	0.11 ± 0.11	0.12 ± 0.10	0.52
Range	0.00, 0.30	0.00, 0.30	
**60 cm-LogMAR UNVA**			
Mean ± SD	0.27 ± 0.16	0.26 ± 0.68	0.25
Range	0.10, 0.50	0.10, 0.40	
**30 cm-LogMAR UNVA**			
Mean ± SD	0.48 ± 0.17	0.43 ± 0.78	0.24
Range	0.10, 0.50	0.10, 0.50	
**Sphere (D)**			
Mean ± SD	−0.30 ± 0.60	−0.31 ± 0.50	0.66
Range	−1.50, 0.75	−1.25, 1.00	
**Cylinder (D)**			
Mean ± SD	−0.60 ± 0.29	−1.18 ± 0.47	< 0.001*
Range	−1.00, 0.00	−2.25, −0.50	
WTR	12 (42.86%)	12 (40.00%)	
ATR	15 (53.57%)	12 (40.00%)	
OA	1 (3.57%)	6 (20.00%)	
**Keratometric astigmatism (D)**			
Mean ± SD	0.59 ± 0.28	1.15 ± 0.45	< 0.001*
Range	0.25, 0.75	0.50, 2.50	

OCCI, opposite clear corneal incision; SCCI, single clear corneal incision; UDVA, uncorrected distance visual acuity; SD, standard deviation; CDVA, corrected distance visual acuity; UNVA, uncorrected near visual acuity; WTR, with the rule; ATR, against the rule; OA, oblique astigmatism.

*p-value < 0.05.

As for manifest refractive outcomes and anterior corneal curvature, the OCCI group had significantly lower postoperative manifest astigmatism (−0.60 ± 0.29 D vs. −1.18 ± 0.47 D, *P* < 0.01) and anterior corneal keratometric astigmatism (0.59 ± 0.28 D vs. 1.15 ± 0.45 D, *P* < 0.01). However, there was virtually no difference in the residual spherical refractive error between the 2 groups (Table 2).

### 3.3 Vector analysis and residual astigmatism distribution

Manifest astigmatism was analyzed by vector analysis (Table 3). The DV in the OCCI group was less than that in the SCCI group (0.59 ± 0.29 vs. 1.15 ± 0.46, *p* < 0.001). The AoE and absolute AoE in the OCCI group (3.17 ± 17.25° and 8.82 ± 11.33°) were also less than those in the SCCI group (−12.17 ± 34.10° and 20.52 ± 22.86°). The differences between the two groups were statistically significant (*p* = 0.04 and *p* = 0.004, respectively). The IoS in the OCCI group was less than that in the SCCI group (0.60 ± 0.22 vs. 1.10 ± 0.37, *P* < 0.001). However, the differences in the SIA, ME and CI between the two groups were not considered statistically significant (*p* = 0.17, 0.16, and 0.08, respectively).

**TABLE 3:** Vector analysis of corneal astigmatism.

Parameter	OCCI group	SCCI group	*p*
**TIA (D)**			
Mean ± SD	1.02 ± 0.49	1.11 ± 0.47	0.32
Range	0.47, 2.09	0.48, 2.06	
**SIA (D)**			
Mean ± SD	0.80 ± 0.70	1.21 ± 1.06	0.16
Range	0.16, 2.65	0.02, 3.70	
**DV (D)**			
Mean ± SD	0.59 ± 0.29	1.15 ± 0.46	< 0.001*
Range	0.00, 1.01	0.49, 2.17	
**ME (D)**			
Mean ± SD	−0.22 ± 0.53	0.10 ± 0.88	0.17
Range	−1.01, 0.94	−1.30, 1.69	
**AoE (degree)**			
Mean ± SD	3.17 ± 17.25	−12.17 ± 34.10	0.04*
Range	−42.83, 39.03	−76.32, 44.50	
**Absolute AoE (degree)**			
Mean ± SD	8.82 ± 11.33	20.52 ± 22.86	0.004*
Range	0.00, 42.83	0.00, 76.32	
**IoS**			
Mean ± SD	0.60 ± 0.22	1.10 ± 0.37	< 0.001*
Range	0.00, 1.04	0.70, 2.33	
**CI**			
Mean ± SD	0.73 ± 0.42	1.06 ± 0.69	0.08
Range	0.30, 1.63	0.02, 2.38	

OCCI, opposite clear corneal incision; SCCI, single clear corneal incision; TIA, Target induced astigmatism; SD, standard deviation; SIA, surgically induced astigmatism; DV, difference vector; ME, magnitude of error; AoE, angle of error; IoS, index of success; CI, correction index.

*p-value < 0.05.

**FIGURE 2:**
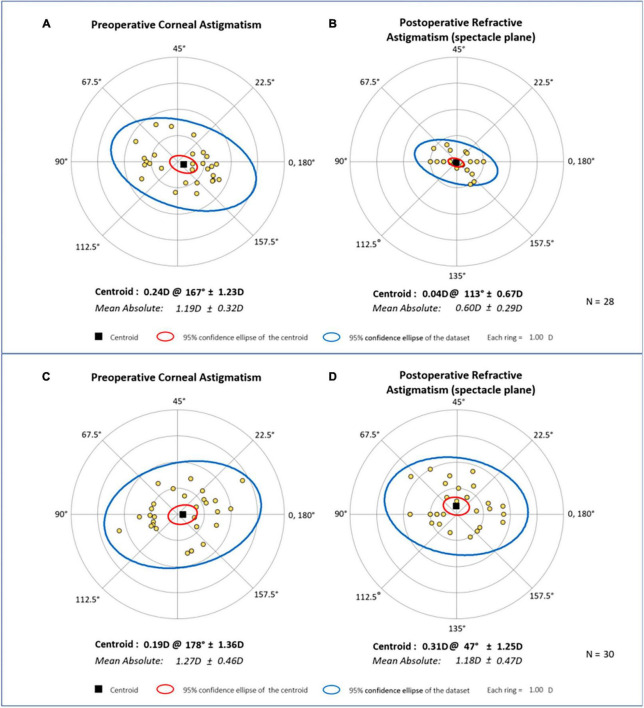
Astigmatism double angle plot, prior to and following surgery. The 95% confidence ellipse area of astigmatism distribution was similar in the OCCI group **(A)** and the SCCI group **(C)** preoperatively. Three months after surgery, the 95% confidence ellipse area of astigmatism distribution in the OCCI group **(B)** decreased significantly, while the SCCI group **(D)** remained similar to its preoperative distribution. OCCI, opposite clear corneal incision; SCCI, single clear corneal incision.

We also analyzed the distribution of residual astigmatism of the two groups. When compared with the SCCI group, residual astigmatisms of the OCCI group were more centralized in the double angle plot (Figures 2B, D). In the OCCI group, the 95% CI of astigmatism distribution reduced noticeably after surgery, and the astigmatism axial distribution was similar to the preoperative state (Figures 2A, B). In the SCCI group, however, the 95% CI of distribution was similar to the preoperative state, and the axial distribution changed evidently after surgery (Figures 2C, D).

**FIGURE 3:**
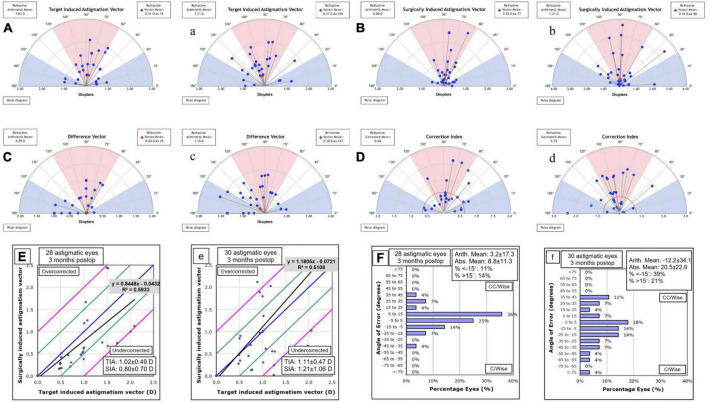
Vector analysis of corneal astigmatism in the OCCI group **(A,B,C,D,E,F)** and the SCCI group **(a,b,c,d,e,f)**. The astigmatism axis distribution of surgically induced astigmatism (SIA) was more similar to target induced astigmatism (TIA) in the OCCI group than in the SCCI group **(A,a)**. Additionally, the distributions of difference vector (DV) and correction index (CI) were closer to the preferred values (i.e., 0 and 1, respectively) in the OCCI group compared to the SCCI group **(C,c)**. The scatterplots of TIA against SIA indicate that the OCCI group were more predictable than the SCCI group **(E,e)**. As for the angle of error, it was within the range of ± 15° for 75% of eyes in the OCCI group, while it was only 39% in the SCCI group **(F,f)**. OCCI, opposite clear corneal incision; SCCI, single clear corneal incision.

Astigmatic measures of vector analyze were shown in Figures 3A–D for OCCI group and a, b, c, d for SCCI group. In addition, we found that the slope was smaller in the OCCI group than in the SCCI group in the scatter diagram of TIA & SIA (Figures 3E, e). In the OCCI group, the angle of error was within ± 5° in 25% of eyes, and within ± 15° in 75% of eyes, compared to 18 and 39% in the SCCI group, respectively (Figures 3F, f).

## 4 Discussion

In the current study, we found that the opposite clear corneal incision (OCCI) effectively reduces residual astigmatism following multifocal IOL implantation. Patients receiving OCCI exhibited desirable visual acuity outcomes and superior refractive profiles compared to those with conventional incisions.

Modern cataract surgery strives to achieve spectacle-free vision, liberating patients from the constraints of cumbersome eyewear such as thick glasses and bifocals. This ultimately translates into enhanced comfort, convenience, and visual clarity. Correcting presbyopia and astigmatism is crucial for this objective.

The IOLs (Lentis Mplus LS-313 MF15) used in the study feature a rotational asymmetry design with a near add of +1.50 D. The design’s advantage lies in its ability to provide optimal visual outcomes at near to intermediate distances without compromising distance visual acuity, additionally exhibiting a low incidence of photic phenomena (4–8). We found that the patients in both groups had relatively good uncorrected distant, intermediate visual acuity and acceptable near vision (Table 2). This significantly reduces their reliance on spectacles. Throughout the follow-up period, no patients reported glare, halos, dysphotopsia or other postoperative optical symptoms.

**FIGURE 4:**
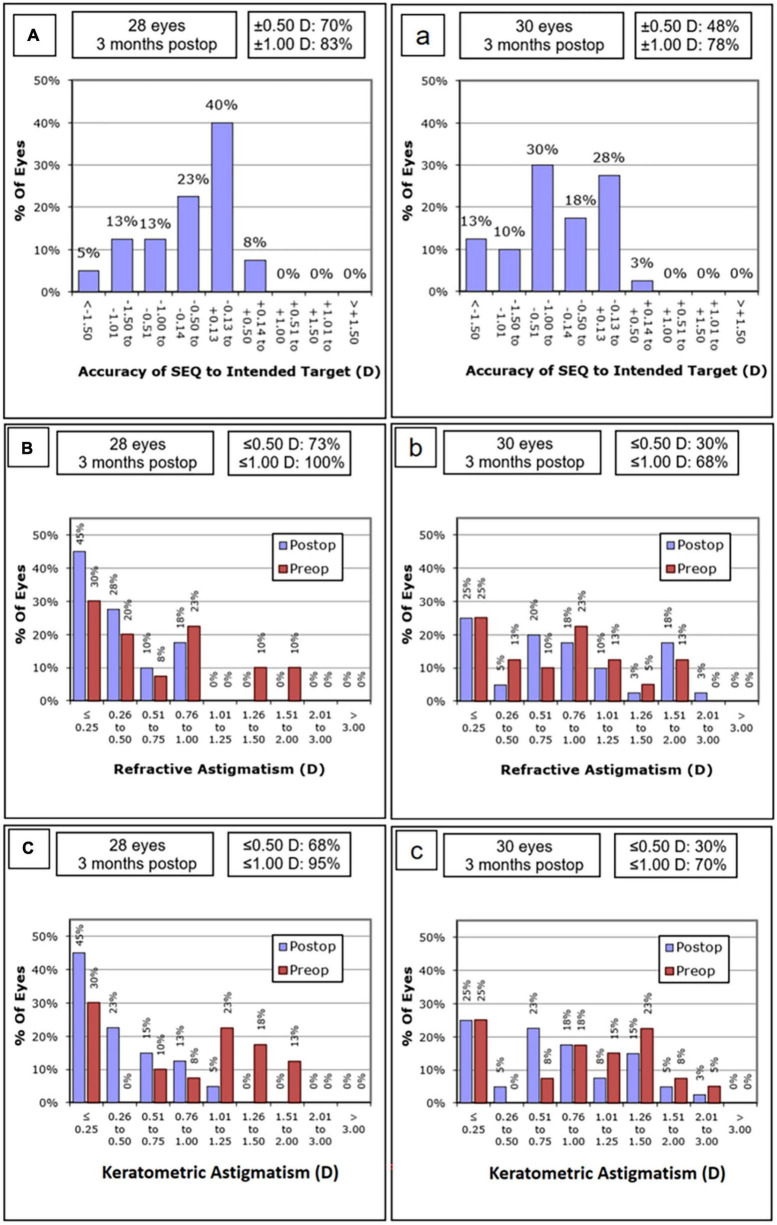
Refractive and keratometric outcomes of the OCCI group **(A,B,C)** and SCCI group **(a,b,c)**. The percentage of eyes achieving the ± 0.50 D target spherical equivalent refraction (SEQ) was 70%, and the ± 1.00 D target was 83% in the OCCI group **(A)**, indicating greater satisfaction compared to the SCCI group **(a)**. In the OCCI group, astigmatism decreased significantly following surgery in both manifest refractive **(B)** and keratometric outcomes **(C)**, compared to the SCCI group, which showed no changes **(b,c)**. OCCI, opposite clear corneal incision; SCCI, single clear corneal incision.

In the OCCI group, placing incisions at the steep keratometry meridian significantly reduced the residual astigmatism compared to the SCCI group. Two-thirds of the eyes in the SCCI group had postoperative astigmatism exceeding 0.5 D, with one-third exceeding 1.0 D. Conversely, three-fourths of OCCI eyes had postoperative astigmatism below 0.5 D, with none exceeding 1.0 D (Figure 4). Vector analysis further support OCCI’s efficacy. Both difference vector (DV) and index of success (IoS) were statistically significantly smaller in the OCCI group, and the polar plots showed a more centralized distributions of astigmatic indices. These findings align with studies using similar incision techniques for monofocal IOLs (22–24), confirming the efficacy of corneal curvature optimized incision planning with multifocal IOLs. Combined with the astigmatism analysis, OCCI emerges as a readily available and cost-effective alternative to expensive toric multifocal IOLs for mild to moderate astigmatism correction.

Intriguingly, the improved astigmatism correction observed with the OCCI did not translate directly into a difference in uncorrected visual acuity (UCVA) across distance, intermediate and near distances between the two groups (Table 2). This discrepancy between astigmatic and visual outcomes can be attributed to several factors.

First, while the difference in residual astigmatism between the two groups was statistically significant, it was relatively small (only around 0.5 D). Moreover, approximately 40% of the eyes in both groups exhibited with-the-rule (WTR) astigmatism postoperatively. It has been reported that cylindrical correction offers limited visual acuity improvement for WTR astigmatism when the magnitude of astigmatism is lower than −0.75 D (25). The combination of the relatively small astigmatic reduction and the mixed effects of astigmatic orientation may partly explain the comparable visual outcomes.

Second, patients with multifocal IOLs demonstrate some tolerance for residual astigmatism (12). And it is particularly true for low levels of astigmatism. Hayashi observed that when astigmatism was less than 1.0 D, eyes implanted with a multifocal IOLs performed better in terms of the visual acuity at both distance and near (26). Additionally, studies have suggested that a lower IOL addition shows better astigmatic defocus tolerability (27, 28). The optical design of IOLs may also influence astigmatic tolerance. McNeely reported high tolerance for residual astigmatism with the Mplus LS-312 MF30, which shares the rotationally asymmetric multifocal design as the MF15 IOL used in the current study, albeit with a higher IOL addition (29).

Third, the current study only measured visual acuity as visual outcomes, which may not fully capture the impact of astigmatism on other aspects of visual performance, such as contrast sensitivity, functional vision, and patient satisfaction (12, 26, 29). Other factors can also affect visual acuity after multifocal IOL implantation, including high order aberrations (30). This may further explain why a significant difference was observed in astigmatism correction but not in visual acuity. Further studies examining these parameters are warranted to fully evaluate the relationship between residual astigmatism and overall visual wellbeing in patients with multifocal IOLs.

The limitations of this study should also be mentioned here. Firstly, due to the retrospective non-randomized design, although the baseline characteristics were balanced between the two groups, potential confounders cannot be fully controlled for. Secondly, only anterior corneal curvature was assessed in the study. The posterior curvature is also an important component of corneal refraction. However, the keratometric astigmatism was consistent with the manifest refractive measurements, both prior to and following surgery. Thirdly, as mentioned earlier, only the visual acuity and refractive outcomes were measured and analyzed in the present study, in addition to the relatively shorter follow-up, further studies should be conducted to include other visual-related measurements, such as contrast sensitivity, wavefront parameters, and patient satisfaction or scales of vision-related quality of life.

In conclusion, the current study confirms that incisions placing at steep K meridian can safely reduce residual astigmatism after cataract surgery. Corneal curvature optimized incision planning, specifically opposite clear corneal incision, combined with rotationally asymmetric multifocal intraocular lens implantation provides optimal visual and refractive outcomes.

## Data availability statement

The raw data supporting the conclusions of this article will be made available by the authors, without undue reservation.

## Ethics statement

The studies involving humans were approved by the Review Board of Zhongshan Hospital (Xiamen), Fudan University. The studies were conducted in accordance with the local legislation and institutional requirements. The participants provided their written informed consent to participate in this study.

## Author contributions

XQ: Conceptualization, Data curation, Formal analysis, Investigation, Methodology, Project administration, Resources, Software, Supervision, Validation, Visualization, Writing – original draft, Writing – review & editing. PY: Conceptualization, Data curation, Formal analysis, Funding acquisition, Investigation, Methodology, Project administration, Resources, Software, Supervision, Validation, Visualization, Writing – review & editing. XW: Conceptualization, Data curation, Formal analysis, Investigation, Methodology, Software, Supervision, Writing – review & editing. YW: Formal analysis, Funding acquisition, Project administration, Resources, Validation, Visualization, Writing – review & editing. YH: Conceptualization, Data curation, Investigation, Methodology, Software, Supervision, Writing – review & editing. ZC: Conceptualization, Data curation, Investigation, Methodology, Software, Supervision, Writing – review & editing. YY: Conceptualization, Data curation, Formal analysis, Funding acquisition, Investigation, Methodology, Project administration, Resources, Software, Supervision, Validation, Visualization, Writing – original draft, Writing – review & editing.

## Footnotes

1
https://calc.apacrs.org/barrett_universal2105

2
https://www.aao.org/calculator

## References

[1] Rosen E Ali JL Dick HB Dell S Slade S. Efficacy and safety of multifocal intraocular lenses following cataract and refractive lens exchange: Metaanalysis of peer-reviewed publications. *J Cataract Refract Surg.* (2016) 42:310–28. 10.1016/j.jcrs.2016.01.014 27026457

[2] Mencucci R Favuzza E Ribeiro F. Editorial: Addressing the unmet needs of cataract patients: When quality of vision can make the difference in quality of life. *Front Med.* (2023) 10:1232243. 10.3389/fmed.2023.1232243 37457565 PMC10344353

[3] Alio JL Plaza-Puche AB Javaloy J Ayala M Moreno LJ Piñero DP. Comparison of a new refractive multifocal intraocular lens with an inferior segmental near add and a diffractive multifocal intraocular lens. *Ophthalmology.* (2012) 119:555–63. 10.1016/j.ophtha.2011.08.036 22218147

[4] Ali JL Plaza-Puche AB Piñero DP Javaloy J Ayala MJ. Comparative analysis of the clinical outcomes with 2 multifocal intraocular lens models with rotational asymmetry. *J Cataract Refract Surg.* (2011) 37:1605–14. 10.1016/j.jcrs.2011.03.054 21855760

[5] Ali JL Piñero DP Plaza-Puche AB Chan MJR. Visual outcomes and optical performance of a monofocal intraocular lens and a new-generation multifocal intraocular lens. *J Cataract Refract Surg.* (2011) 37:241–50. 10.1016/j.jcrs.2010.08.043 21241905

[6] Berrow EJ Wolffsohn JS Bilkhu PS Dhallu S. Visual performance of a new bi-aspheric, segmented, asymmetric multifocal IOL. *J Refract Surg.* (2014) 30:584–8. 10.3928/1081597X-20140814-01 25250414

[7] Vargas V Ferreira R Barrio JLAD Ali JL. Visual outcomes, patient satisfaction, and light distortion analysis after blended implantation of rotationally asymmetric multifocal intraocular lenses. *J Refract Surg.* (2020) 36:796–803. 10.3928/1081597X-20200902-01 33295991

[8] Oshika T Arai H Inoue Y Fujita Y. Five-year clinical outcomes of low-add-power segmented rotationally asymmetrical intraocular lens. *Ophthalmol Ther.* (2023) 12:1649–56. 10.1007/s40123-023-00703-2 36947345 PMC10164212

[9] Guan Z Yuan F Yuan YZ Niu WR. Analysis of corneal astigmatism in cataract surgery candidates at a teaching hospital in Shanghai, China. *J Cataract Refract Surg.* (2012) 38:1970–7. 10.1016/j.jcrs.2012.07.025 23079313

[10] Day AC Dhariwal M Keith MS Ender F Vives CP Miglio C Distribution of preoperative and postoperative astigmatism in a large population of patients undergoing cataract surgery in the UK. *Br J Ophthalmol.* (2019) 103:993–1000. 10.1136/bjophthalmol-2018-312025 30190365 PMC6591741

[11] Lundström M Dickman M Henry Y Manning S Tassignon MJ Young D Risk factors for refractive error after cataract surgery: Analysis of 282 811 cataract extractions reported to the European registry of quality outcomes for cataract and refractive surgery. *J Cataract Refract Surg.* (2018) 44:447–52. 10.1016/j.jcrs.2018.01.031 29685779

[12] Schallhorn SC Hettinger KA Pelouskova M Teenan D Venter JA Hannan SJ Effect of residual astigmatism on uncorrected visual acuity and patient satisfaction in pseudophakic patients. *J Cataract Refract Surg.* (2021) 47:991–8. 34290195 10.1097/j.jcrs.0000000000000560

[13] Shen W Zhuo B Zhang L Shen J Ma D Yang J. Effect of astigmatism on visual outcomes after multifocal intraocular lens implantation: A systematic review and meta-analysis. *Front Med.* (2023) 10:1214714. 10.3389/fmed.2023.1214714 38089878 PMC10713711

[14] Rubenstein JB Raciti M. Approaches to corneal astigmatism in cataract surgery. *Curr Opin Ophthalmol.* (2013) 24:30–4. 23197264 10.1097/ICU.0b013e32835ac853

[15] Rocha-de-Lossada C Rodríguez-Vallejo M Rodríguez-Calvo-de-Mora M Ribeiro FJ Fernández J. Managing low corneal astigmatism in patients with presbyopia correcting intraocular lenses: A narrative review. *BMC Ophthalmol.* (2023) 23:254. 10.1186/s12886-023-03003-2 37280550 PMC10243013

[16] D’Oria F Alio JL Martinez-Abad A Luis I Pablo L Abdelghany AA. Astigmatic change as a predictor of intrastromal corneal ring segment late extrusion. *J Cataract Refract Surg.* (2022) 48:401–7. 10.1097/j.jcrs.0000000000000774 34393182

[17] Rosen ES. Opposite clear corneal incisions. *J Cataract Refract Surg.* (2000) 26:789–90. 10889411 10.1016/s0886-3350(00)00503-4

[18] Mendicute J Irigoyen C Ruiz M Illarramendi I Ferrer-Blasco T Montés-Mic R. Toric intraocular lens versus opposite clear corneal incisions to correct astigmatism in eyes having cataract surgery. *J Cataract Refract Surg.* (2009) 35:451–8. 19251137 10.1016/j.jcrs.2008.11.043

[19] Ren Y Fang X Fang A Wang L Jhanji V Gong X. Phacoemulsification with 3.0 and 2.0 mm opposite clear corneal incisions for correction of corneal astigmatism. *Cornea.* (2019) 38:1105–10. 30844842 10.1097/ICO.0000000000001915

[20] Tadros A Habib M Tejwani D Lany H Thomas P. Opposite clear corneal incisions on the steep meridian in phacoemulsification: Early effects on the cornea. *J Cataract Refract Surg.* (2004) 30:414–7. 10.1016/S0886-3350(03)00649-7 15030833

[21] Alpins N. Astigmatism analysis by the Alpins method. *J Cataract Refract Surg.* (2001) 27:31–49. 11165856 10.1016/s0886-3350(00)00798-7

[22] Binayi Faal N Ojaghi H Sadeghieh Ahari S. Paired opposite 4 mm clear corneal incisions on steep meridian during phacoemulsification. *J Curr Ophthalmol.* (2021) 33:400–7. 10.4103/joco.joco_205_20 35128185 PMC8772503

[23] Razmjoo H Koosha N Vaezi MH Rahimi B Peyman A. Corneal astigmatism change and wavefront aberration evaluation after cataract surgery: “Single” versus “paired opposite” clear corneal incisions. *Adv Biomed Res.* (2014) 3:163. 10.4103/2277-9175.139126 25221766 PMC4162035

[24] Vats S Kumari L Goenka R Agrawal M Mishra S. Pattern of astigmatism using partial coherence interferometry in patients of different age groups undergoing cataract surgery. *Oman J Ophthalmol.* (2022) 15:295–8. 10.4103/ojo.ojo_345_21 36760961 PMC9905882

[25] Tan QQ Wen BW Liao X Tian J Lin J Lan CJ. Optical quality in low astigmatic eyes with or without cylindrical correction. *Graefes Arch Clin Exp Ophthalmol.* (2020) 258:451–8. 31641885 10.1007/s00417-019-04501-0

[26] Hayashi K Hayashi H Nakao F Hayashi F. Influence of astigmatism on multifocal and monofocal intraocular lenses. *Am J Ophthalmol.* (2000) 130:477–82. 11024420 10.1016/s0002-9394(00)00526-2

[27] Altinkurt E Muftuoglu O. Comparison of three different diffractıve multifocal intraocular lenses with a +2.5, +3.0, and +3.75 diopter additıon power. *Saudi J Ophthalmol.* (2019) 33:353–62. 10.1016/j.sjopt.2019.09.007 31933530 PMC6950979

[28] Carones F. Residual astigmatism threshold and patient satisfaction with bifocal, trifocal and extended range of vision intraocular lenses (IOLs). *Open J Ophthalmol.* (2017) 7:1–7.

[29] McNeely RN Pazo E Millar Z Richoz O Nesbit A Moore TCB Threshold limit of postoperative astigmatism for patient satisfaction after refractive lens exchange and multifocal intraocular lens implantation. *J Cataract Refract Surg.* (2016) 42:1126–34. 10.1016/j.jcrs.2016.05.007 27531287

[30] Alio JL D’Oria F Toto F Balgos J Palazon A Versaci F Retinal image quality with multifocal, EDoF, and accommodative intraocular lenses as studied by pyramidal aberrometry. *Eye Vis.* (2021) 8:37. 10.1186/s40662-021-00258-y 34615549 PMC8496005

